# Supplementary material for: Kidney damage and associated risk factors in the rural Eastern Cape, South Africa: A cross-sectional study
Source: PLoS One. 2024 Sep 9;19(9):e0292416. doi: 10.1371/journal.pone.0292416 (PMC11383248; doi:10.1371/journal.pone.0292416)
Supplement: Attachment — Submitted filename: Review PONE-D-23-27552.docx [file pone.0292416.s001.docx]

This observational study investigates the prevalence of kidney damage within the rural community of the Eastern Cape province in South Africa. Notably, the research delves into the association of kidney damage with various risk factors linked to Chronic Kidney Disease (CKD). This study serves as a crucial endeavor in understanding the prevalence of CKD within the context of rural South Africa.

However, upon careful review, certain aspects require further elucidation and refinement.

1. Firstly, it is imperative to delineate the relationship between kidney function and specific factors, such as diabetes, hypertension, and proteinuria, differentiating the data based on gender. Analyzing these associations separately for males and females could provide a more comprehensive understanding of the impact of these factors on kidney health.
2. Moreover, the report highlights that 67 patients were classified between G3-G5 stages of CKD. However, the distribution of these patients in terms of gender remains ambiguous. It is essential to clarify the proportion or percentage of male and female patients within these stages for a more nuanced interpretation of the data.
3. Additionally, exploring the relationship between the grades of CKD and associated risk factors might offer deeper insights into the progression of the disease and its underlying causes. By examining how different risk factors correspond with the severity of CKD, a clearer understanding of the disease's trajectory can be obtained.
4. In reference to Table 2, while the data for Female CKD (49) and No-CKD (223) is presented, the information pertaining to males seems to be absent. Clarification is required regarding whether Table 2 is exclusively related to female subjects or if the data for male subjects was inadvertently omitted.
5. Lastly, in the paragraph discussing the 'Associations of CKD with potential risk factors,' an apparent inconsistency is noted regarding the relationship between age and the incidence of CKD. Contrary to the initial suggestion that the incidence of CKD declines with age, Figure 1 clearly illustrates an increasing ratio of CKD to Non-CKD cases with advancing age. This inconsistency should be rectified to ensure accurate interpretation and reporting of the data.
