# Supplementary material for: Kidney damage and associated risk factors in the rural Eastern Cape, South Africa: A cross-sectional study
Source: PLoS One. 2024 Sep 9;19(9):e0292416. doi: 10.1371/journal.pone.0292416 (PMC11383248; doi:10.1371/journal.pone.0292416)
Supplement: Attachment — Submitted filename: REBUTTAL Gonzalez.docx [file pone.0292416.s002.docx]

Dear Editor,

Thanks for the insightful comments of the reviewers. Indeed, the manuscript has improved remarkably with the suggestions and queries from the reviewers. Please find below the responses to the reviewers’ comments.

Regards

Ernesto Rosales Gonzalez

Reviewer #1: 
1. It is necessary to show in more detail the clinical and social background of the regions.

Response: Thanks for the comment. The clinical and social background has been included from lines 54 to 61 and 88 to 95.

2. It is necessary to make comparisons with other regions and clarify the characteristics of the target region.

Response: Thanks for the comment. We have added more information on the study setting.

3. Information about treatment for HT, DM and CKD is needed.

Response: Thanks for the comment. We have made corrections

Reviewer #2:

1. The authors investigated the prevalence of CKD in the Rural Eastern Cape, South Africa. Although the findings were partially meaningful, there are several serious problems. Especially, it is not surprising that both older age and the presence of proteinuria are strongly associated with the presence of CKD. Although they aimed to clarify the prevalence of CKD in this area, they included only outpatients of the hospitals.

Response: Thanks for the comment. It should be clarified that the study comprised of stable patients attending the primary health care outpatient setting. This is the first point of contact with patients in the rural South African settings. More studies are needed to elucidate on the in-patient in rural hospital settings in South Africa.

2. In contrast, the study by Adeniyi AB et al. [Ref. 9] included young school teachers, and the prevalence of CKD was lower as compared to this study. From this study design, they cannot conclude that early detection of proteinuria and decreased renal function could lead to prompt initiation of preventative measures and management to delay the progression to end-stage kidney failure and mortality.

Response: Thanks for the insight. We have rephrase our conclusion on this.

Abstract
P.2, L.15: The authors described that significant kidney damage was defined as low eGFR (<60mL/min per 1.73m2) and/or the presence of proteinuria. However, the chief outcome of this study seems to be the prevalence of CKD defined by KDIGO Guideline.

Response: Thanks for the comment. We have made corrections

P.2, L.21: Although the authors described that older age and the presence of proteinuria were associated with the significant kidney damage, both ORs were less than 1.
Response: Thanks for the comment. We have made corrections.

Introduction
P.3, L.30-32: Abbreviations of RRT should be spelled out at first usage and the abbreviated form used thereafter. Please check all abbreviations including CKD, eGFR, CKD-EPIcreatinine or MDRD.
Response: Thanks for the comment. We have made corrections as suggested

Methods
P.8, L.133: Ref.14 is guideline for the management of glomerular diseases. CKD should be defined by KDIGO guideline [Levey AS, et al. Kidney Int 2005；67：2089‒100].
Response: Thanks for the comment. We have made corrections as suggested

Results
P.9, L.156 and Table 1: The authors described that ten participants reported prior diagnosis of CKD at the time of the study (Table 1). However, there was no description in Table 1. There is no need to present the number and proportion of both female and male participants. Not-normally distributed variables, especially serum creatinine and urine protein, should be presented as medians and ranges or interquartile ranges (IQR).
Response: Thanks for the comment. We have made corrections as suggested

P.11, Table 2: The number of HIV patients without CKD is odd. Was the prevalence of CKD higher among HIV patients? Urine protein of CKD and non-CKD were odd.
Response: Thanks for the comment. We have made corrections as suggested

P.12, Table 3: ORs of high age and urine protein should be more than 1. P-value of diabetic mellitus is odd. Although the authors described the abbreviations of HTN, DM, eGFR and CKD-EPI below the Table 3, they did not use in the Table.
Response: Thanks for the comment. We have made corrections as suggested

P.13, Table 4: The data of both patients with urine protein >30 and those with urine protein<30 should be presented. Although the OR of HIV is 0.46 (0.21-1.01), p-value was less than 0,05. OR of triglyceride is odd.
Response: Thanks for the comment. We have made corrections as suggested

P.14, Table 5: The objective variable of this multivariate analysis is unclear. It is not much meaning to find that hypertension and higher serum creatinine level were associated with higher prevalence of proteinuria.

Response: Thanks for the comment. We have made corrections as suggested

Reviewer #3:

This cross-sectional study describes the prevalence of CKD in patients attending an outpatient clinic of a rural community in the eastern cape of South Africa. The article is well written and easy to understand. There are some limitations to the study including the homogeneity of the study population, the fact that this study was conducted in patients attending the out-patient department of a community clinic but these have been mentioned by the authors.
There are a few clarifications which should be addressed.

1. Please describe the outpatient department in more detail. Does it provide wellness and preventive services or it caters to patients who are being followed up for several illnesses? Although the results suggest the latter, this should be clearly stated in the methodology.

Response: Thanks for the comment. We have made corrections
2. References are not always well written, some references do not have year of publications, and different styles have been used. Please refer to Plos one referencing style.

Response: Thanks for the comment. We have made corrections as suggested

3. Some of the references do not seem relevant to the text, for example ref 19 refers to prevalence of CKD in Cameroonians. References should be sought from South African patients while discussing the demographics.
Reference 27 refers to precision nephrology, it may be better to quote the article quoted in that write-up: Minutolo R et al... Sex differences in the progression of CKD.......
In Conclusion, the study adds to the body of knowledge on prevalence of CKD to a subset of South Africans. Minor corrections noted above should be made.

Response: Thanks for the comment. We have made corrections as suggested.

Reviewer #4:

This observational study investigates the prevalence of kidney damage within the rural community of the Eastern Cape province in South Africa. Notably, the research delves into the association of kidney damage with various risk factors linked to Chronic Kidney Disease (CKD). This study serves as a crucial endeavor in understanding the prevalence of CKD within the context of rural South Africa.
However, upon careful review, certain aspects require further elucidation and refinement.

1. Firstly, it is imperative to delineate the relationship between kidney function and specific factors, such as diabetes, hypertension, and proteinuria, differentiating the data based on gender. Analyzing these associations separately for males and females could provide a more comprehensive understanding of the impact of these factors on kidney health.

Response: Thanks for the comment. We have made corrections as suggested

2. Moreover, the report highlights that 67 patients were classified between G3-G5 stages of CKD. However, the distribution of these patients in terms of gender remains ambiguous. It is essential to clarify the proportion or percentage of male and female patients within these stages for a more nuanced interpretation of the data.

Response: Thanks for the comment. We have made corrections as suggested

3. Additionally, exploring the relationship between the grades of CKD and associated risk factors might offer deeper insights into the progression of the disease and its underlying causes. By examining how different risk factors correspond with the severity of CKD, a clearer understanding of the disease's trajectory can be obtained.

Response: Thanks for the comment. We have made corrections as suggested

4. In reference to Table 2, while the data for Female CKD (49) and No-CKD (223) is presented, the information pertaining to males seems to be absent. Clarification is required regarding whether Table 2 is exclusively related to female subjects or if the data for male subjects was inadvertently omitted.

Response: Thanks for the comment. We have made corrections as suggested

5. Lastly, in the paragraph discussing the 'Associations of CKD with potential risk factors,' an apparent inconsistency is noted regarding the relationship between age and the incidence of CKD. Contrary to the initial suggestion that the incidence of CKD declines with age, Figure 1 clearly illustrates an increasing ratio of CKD to Non-CKD cases with advancing age. This inconsistency should be rectified to ensure accurate interpretation and reporting of the data.

Response: Thanks for the comment. We have made corrections as suggested

Reviewer #5:

This is an interesting study as very few data exist about CKD in rural areas of South Africa. The authors should be congratulated for their efforts to investigate CKD epidemiology in a region with shortages of health facilities.
However, I do have some comments.

1. The authors should give more information about the region. How much remoted is this? What are the main demographic data of the whole population and study group (employment, length of life, poverty, educational status, access to health facilities and so on.

Response: Thanks for the comment. We have made corrections as suggested.

2. There are no data about smoking and alcohol.

Response: Thanks for the comment. We have made corrections as suggested

3. There is a possibility of selection bias as the participants were outpatients of the Health Centre. The authors should comment on this.

Response: Thanks for the comment. This primary health care study is the first in the Eastern Cape province, however, future studies should focus on CKD at the community level.

4. The authors should give a chart about the initial number of persons asked to participate. How many people refused or were excluded?

Response: Thanks for the comment. We have made corrections as suggested

5. I miss information about the questionnaire given to patients and how it was filled in (assisted? , could all patients read?)

Response: Thanks for the comment. The research nurse provided assistance to the participants when required (line 107)

6. In the discussion, the authors could add comments/comparisons with other South African rural areas data ( Navise et al. <https://doi.org/10.1186/s12882-023-03068-7> June Fabian et al <https://doi.org/10.12688/wellcomeopenres.18016.2>).

Response: Thanks for the comment. We have made corrections as suggested

This observational study investigates the prevalence of kidney damage within the rural community of the Eastern Cape province in South Africa. Notably, the research delves into the association of kidney damage with various risk factors linked to Chronic Kidney Disease (CKD). This study serves as a crucial endeavor in understanding the prevalence of CKD within the context of rural South Africa. However, upon careful review, certain aspects require further elucidation and refinement.

1. Firstly, it is imperative to delineate the relationship between kidney function and specific factors, such as diabetes, hypertension, and proteinuria, differentiating the data based on gender. Analyzing these associations separately for males and females could provide a more comprehensive understanding of the impact of these factors on kidney health.

Response: Thanks for the comment. We have made corrections as suggested

2. Moreover, the report highlights that 67 patients were classified between G3-G5 stages of CKD. However, the distribution of these patients in terms of gender remains ambiguous. It is essential to clarify the proportion or percentage of male and female patients within these stages for a more nuanced interpretation of the data.

Response: Thanks for the comment. We have made corrections as suggested

3. Additionally, exploring the relationship between the grades of CKD and associated risk factors might offer deeper insights into the progression of the disease and its underlying causes. By examining how different risk factors correspond with the severity of CKD, a clearer understanding of the disease's trajectory can be obtained.

Response: Thanks for the comment. We have made corrections as suggested

4. In reference to Table 2, while the data for Female CKD (49) and No-CKD (223) is presented, the information pertaining to males seems to be absent. Clarification is required regarding whether Table 2 is exclusively related to female subjects or if the data for male subjects was inadvertently omitted.

Response: Thanks for the comment. We have made corrections as suggested

5. Lastly, in the paragraph discussing the 'Associations of CKD with potential risk factors,' an apparent inconsistency is noted regarding the relationship between age and the incidence of CKD. Contrary to the initial suggestion that the incidence of CKD declines with age, Figure 1 clearly illustrates an increasing ratio of CKD to Non-CKD cases with advancing age. This inconsistency should be rectified to ensure accurate interpretation and reporting of the data.

Response: Thanks for the comment. We have made corrections as suggested
