# Supplementary material for: Kidney damage and associated risk factors in the rural Eastern Cape, South Africa: A cross-sectional study
Source: PLoS One. 2024 Sep 9;19(9):e0292416. doi: 10.1371/journal.pone.0292416 (PMC11383248; doi:10.1371/journal.pone.0292416)
Supplement: Attachment — Submitted filename: Response letter Editor Reviewers comments.docx [file pone.0292416.s003.docx]

**Reviewer’s comments**

Comment 1: The candidate uploaded the corrected manuscript with Track changes but not to the unmarked manuscript with no Track changes. Table 6 needs to be included in the corrected manuscript, and there was no corresponding correction in the abstract section.

Response: We have provided the marked and clean copies.

Comment 2: Hence, the corrected Manuscript in the unmarked should be uploaded, the table numbering corrected, and the abstract should reflect the changes made in the new tables 8.

Response: Thanks for the suggestions. We also made changes in the abstract.

Comment 3: Abstract: Page 2, Line 22-23 - Based on the changes the authors have effected in the body of the manuscript, the following statement should be corrected: “Risk factors for significant kidney damage were older age (OR=1.08, 95% CI 1.06-1.1, p<0.001)
Specifically, I request that you review the abstract to ensure it accurately reflects the changes made in the manuscript. This includes the correction of the statement regarding the risk factors for significant kidney damage.

Response: We have made changes as suggested.

Comment 4: Page 15, line 289: In line with reviewers' comments, the authors have changed the data points for Age on Table 8 (Multiple regression). Please make the same changes in the text and in the abstract.

Response: We also made changes as suggested.

Comment 5: Similarly, the authors have removed urine protein from the table. Please make the same change in the abstract.
Table 8: Please clarify if the variable ‘Age’ as used in the logistic regression models was a continuous or categorical variable.

Response: Age was used as a continuous variable.

Comment 6: Limitations: Page 20, line 392—Being a facility-based study with respondents recruited from the outpatient clinic limits the ability to generalise the findings to the general population. Please kindly indicate this fact as part of the study limitations.
Response: We have made changes as suggested.

Comment 7: References: Please kindly ensure that all the references are in alignment with the journal requirements
Response: We have made changes as suggested.
